# Postoperative respiratory failure in liver transplantation: Risk factors and effect on prognosis

**DOI:** 10.1371/journal.pone.0211678

**Published:** 2019-02-11

**Authors:** Alfonso Wolfango Avolio, Rita Gaspari, Luciana Teofili, Giuseppe Bianco, Giorgia Spinazzola, Paolo Maurizio Soave, Gianfranco Paiano, Alessandra Gioia Francesconi, Andrea Arcangeli, Nicola Nicolotti, Massimo Antonelli

**Affiliations:** 1 Fondazione Policlinico Universitario A. Gemelli IRCCS, Department of Surgery -Transplantation Service, Rome, Italy; 2 Università Cattolica del Sacro Cuore, Rome, Italy; 3 Fondazione Policlinico Universitario A. Gemelli IRCCS, Department of Anaesthesiology and Intensive Care Medicine, Rome, Italy; 4 Fondazione Policlinico Universitario A. Gemelli IRCCS, Institute of Hematology, Rome, Italy; 5 Fondazione Policlinico Universitario A. Gemelli IRCCS, Institute of Hygiene and Epidemiology, Rome, Italy; Universidad de Navarra, SPAIN

## Abstract

**Background:**

Postoperative respiratory failure (PRF, namely mechanical ventilation >48 hours) significantly affects morbidity and mortality in liver transplantation (LTx). Previous studies analyzed only one or two categories of PRF risk factors (preoperative, intraoperative or postoperative ones).

The aims of this study were to identify PRF predictors, to assess the length of stay (LoS) in ICU and the 90-day survival according to the PRF in LTx patients.

**Methods:**

Two classification approaches were used: systematic classification (recipient-related preoperative factors; intraoperative factors; logistic factors; donor factors; postoperative ICU factors; postoperative surgical factors) and patient/organ classification (patient-related general factors; native-liver factors; new-liver factors; kidney factors; heart factors; brain factors; lung factors). Two hundred adult non-acute patients were included. Missing analysis was performed. The competitive role of each factor was assessed.

**Results:**

PRF occurred in 36.0% of cases. Among 28 significant PRF predictors at univariate analysis, 6 were excluded because of collinearity, 22 were investigated by ROC curves and by logistic regression analysis. Recipient age (OR = 1.05; p = 0.010), female sex (OR = 2.75; p = 0.018), Model for End-Stage Liver Disease (MELD, OR = 1.09; p<0.001), restrictive lung pattern (OR = 2.49; p = 0.027), intraoperative veno-venous bypass (VVBP, OR = 3.03; p = 0.008), pre-extubation PaCO_2_ (OR = 1.11; p = 0.003) and Model for Early Allograft Function (MEAF, OR = 1.37; p<0.001) resulted independent PRF risk factors. As compared to patients without PRF, the PRF-group had longer LoS (10 days IQR 7–18 versus 5 days IQR 4–7, respectively; p<0.001) and lower day-90 survival (86.0% versus 97.6% respectively, p<0.001).

**Conclusion:**

In conclusion, MELD, restrictive lung pattern, surgical complexity as captured by VVBP, pre-extubation PaCO_2_ and MEAF are the main predictors of PRF in non-acute LTx patients.

## Introduction

Postoperative pulmonary complications occur in 5 to 10% of surgical patients, and in 9 to 40% abdominal surgical patients [1–3]. Infectious and non-infectious pulmonary complications are the main cause of early postoperative morbidity, early mortality, and increased hospital stay [1–3]. Postoperative respiratory failure (PRF), defined as the need for mechanical ventilation for more than 48 hours after surgery is among the most serious postoperative pulmonary complications [3–4].

Candidates to liver transplantation (LTx) exhibit several concomitant morbidities and have to face the effects of an extremely invasive surgery, sometimes complicated by massive bleeding. Moreover, the transplant procedure itself implies bilateral transection of the abdominal muscles, insult on chest wall related to retractor, and diaphragmatic impairment [5–9]. Finally, in patients with primary non-function of the graft and related dysfunction of other organs (severe encephalopathy, hemodynamic instability, renal failure), the need for an urgent re-transplant contraindicates the liberation from mechanical ventilation.

Several factors including age, female sex, degree of liver decompensation, previous lung abnormalities, renal impairment, diabetes, and preoperative donor data have been variably associated with pulmonary complications or specifically with PRF [6,10–18]. In general, comparison among studies is impaired by the different definitions and timings adopted for endpoint evaluation. In addition, no study has defined the relevance of potential risk factors for PRF by excluding collinearity and Odd Ratios (OR) are available only in few studies [12–16]. Overall, intraoperative surgical complexity has been investigated only in terms of transfusion needs [11,13–16], while the relationship between PRF and postoperative graft recovery has been scarcely explored [11,13,19].

The aim of this study is to investigate risk factors for PRF in LTx patients, as well as the PRF impact on the prognosis.

## Materials and methods

### Study design

This retrospective study was designed to identify potential risk factors for PRF (primary end-point) and to estimate the length of stay (LoS) in ICU and day 90-survival according to PRF (secondary end-points) in LTx patients. Adult patients admitted to the postoperative Intensive Care Unit (ICU) of Fondazione Policlinico Universitario A.Gemelli IRCCS of Rome between January 2010 and August 2017 were included. LoS was calculated as the difference in days from the day of discharge (ICU or hospital) and the day of transplant. Patients who died in ICU or hospital were excluded from LoS calculation. The study was approved by the Institutional Review Board of “Istituto di Anestesiologia e Rianimazione” of Fondazione Policlinico Universitario A.Gemelli IRCCS, Rome, Italy.

### Data

We used a routinely-collected anonymous data set on liver transplants, prospectively gathered at our institution. We counted the number of missing data for each variable and we included only variables with a percentage of missing less than or equal to 8%. Variables relevant to the study purpose are shown in S1 Table. Multivariable analysis was performed on the same number of observations.

Echocardiography, pulmonary function tests, and arterial blood gas analyses were obtained as a part of the routine preoperative evaluation. The pulmonary defect pattern was defined as obstructive in presence of a forced expiratory flow in 1 second / forced vital capacity ratio ≤70% of the predicted value, or as restrictive if the total lung capacity was <80% of the predicted value [20]. Restrictive pattern was defined according to the percentage of predicted total lung capacity: mild (≥70%), moderate (60–69%) and severe (<60%) [20]. Pleural effusion was defined as moderate-severe when the estimation by ultrasonography suggested a volume greater than 500 mL [21]. Ascites was defined as mild (<5 L) or moderate-severe (≥5 L) according to the intraoperative aspiration. Hepatic encephalopathy grade was defined according to West Haven criteria [22]. Intraoperative fluid balance was monitored through Swan-Ganz catheter and esophageal Doppler. Crystalloids were administered (3–5 mL/Kg/h) to maintain a central venous pressure target of 5 mmHg and avoid bleeding from liver bare areas; each major hemodynamic change was counteracted by crystalloids and/or vasoactive drugs administration. Postoperatively, a slightly negative fluid balance was maintained. The value of 9 g/dL of hemoglobin was adopted as target for transfusion.

Patients were considered ready for weaning from mechanical ventilation according to following criteria: hemodynamic stability, body temperature <38° C, pressure support ≤8 cm H_2_O with positive end-expiratory pressure ≤5 cm H_2_O, SaO_2_ >90% on FiO_2_ ≤0.4, respiratory rate ≤35 breaths/min, maximal inspiratory pressure ≤20 cm H_2_O, tidal volume >5 ml/Kg, rapid shallow breathing index <105 breaths/min/L, no respiratory acidosis and adequate level of consciousness [23]. PRF was defined as the need for mechanical ventilation for more than 48 hours after transplantation or the reinstitution of mechanical ventilation (invasive or non-invasive) at any time during the ICU stay after liver transplantation [16]. PRF patients were further grouped as weaning failure (WF) patients if not fulfilled weaning criteria at 48 h after transplant or as extubation failure (EF) patients if they were extubated within 48 hours but required the reinstitution of mechanical ventilation, either through reintubation or non-invasive ventilation, according to the tolerability of non-invasive ventilation interface and gas exchange efficacy [24]. Tracheotomy was performed in patients needing ventilation for more than 10 days. Infectious postoperative pulmonary complications were defined according to the American Society of Infectious Disease guidelines criteria [25].

### Scores

Eleven scores were included in the analyses. Seven scores refer to organ functions and include Model for End-stage Liver Disease (MELD) [26] and MELDNa at listing [27], MELD and MELDNa at transplant, MELD at day 3 post operation (3-pod) [28], Model for Early Allograft Function (MEAF) [29], and Risk Injury Failure Loss End-stage of kidney disease (RIFLE) [30]. Two additional scores, donor age x MELD (D-MELD) [31], and BAlance of Risk [32] regard the match between quality of the donor and disease severity of the patient. One score, the Simplified Acute Physiology Score (SAPS II), refers to a multisystem evaluation of critical patients [33]. The Dindo-Clavien score obtained at hospital discharge [34] was used only to provide an efficacious stratification among groups and subgroups in terms of complication prevalence.

#### Allocation, surgery, graft perfusion and immunosuppressive therapy

Cirrhotic patients were prioritized for transplantation according to the disease severity and ranked by MELD score [26]. Patients with hepatocellular carcinoma (HCC) were equalized to cirrhotic ones according to the Italian allocation system, deserving attention to donor-recipient match [31,35–38]. The operation was performed in order to minimize the ischemia time. The hepatectomy started when the donor team was on the way back. Cases with delay in the graft availability were managed at the end of hepatectomy by VVBP or termino-lateral temporal porto-caval anastomosis. Split livers (n = 2) were performed in situ. The grafts were perfused with University of Wisconsin or Histidine-Tryptophan-Ketoglutarate solution, as previously reported [39]. All patients received immunosuppressive therapy consisting of the combination of calcineurin inhibitors (cyclosporine or tacrolimus), mofetyl-micophenolate and low-dose steroids starting on postoperative day 0. In cases with renal impairment, calcineurin inhibitors’ administration was postponed and introduced at adjusted doses according with renal function recovery. Biopsy proven rejection episodes were treated with steroid boluses and, if resistant, with anti-thymocyte polyclonal globulins.

### Risk assessment

The overall risk was assessed combining risk factors according to a systematic classification and to a patient- and organ- specific classification (Table 1).

**Table 1:** Dual-perspective approach to potential risk factors.

**A. Systematic approach**	Potential risk factors
**A1. Preoperative factors related to** **the recipient**	Age at transplant; Sex; BMI; Indication; Diabetes; MELD at transplant; MELDNa at transplant; Hemofiltration; Left Ventricular Ejection Fraction percentage; Systolic pulmonary artery pressure; Diastolic dysfunction; pH; PaO_2_ and PaCO_2_ at listing; Restrictive or Obstructive pattern at Pulmonary Function Tests; Encephalopathy grade; Hepatopulmonary syndrome; Portopulmonary syndrome
**A2. Intraoperative factors**	VVBP; Porto-caval anastomosis; transfusion requirements (Packet red blood cells, Fresh frozen plasma, Platelets); Operation time
**A3. Logistic factors**	D-MELD; BAR; CIT
**A4. Donor factors**	Donor age; Standard donor/non-standard donor [40]; Extended criteria donor/non-extented criteria donor [41]
**A5. Postoperative ICU factors**	Hemofiltration or Hemodialysis; SAPS II; Mechanical Ventilation; PaO_2;_ PaCO_2;_ PaO_2_/FiO_2_ ratio; Post-operative Pulmonary Complications
**A6. Postoperative surgical factors**	MEAF; MELD at the 3^rd^pod; RIFLE at the 3^rd^pod (2–3 versus 0–1); creatinine at the 3^rd^pod
**B. Patient or organ based approach**	
**B1. General factors (patient)**	Age at transplant; Sex; BMI; Diabetes; SAPS II
**B2. Native-liver factors**	Indication; MELD at listing; MELD at transplant; MELDNa at listing; MELDNa at transplant; Hepatopulmonary syndrome; Portopulmonary syndrome; VVBP; Transfusion requirements (Packet red blood cells, Fresh frozen plasma, Platelets)
**B3. New-liver factors**	Donor Age; Standard donor/non-standard donor [40]; Extended criteria donor/non-extented criteria donor [41]; D-MELD; BAR; MEAF; MELD at the 3^rd^pod; CIT; Operation time
**B4. Kidney factors**	Hemofiltration; RIFLE at the 3^rd^pod; creatinine at the 3^rd^pod
**B5. Heart factors**	Left Ventricular Ejection Fraction percentage; Systolic Pulmonary Artery Pressure; Diastolic dysfunction
**B6. Brain factors**	Encephalopathy grade
**B7. Lung factors**	Mechanical Ventilation; pH, PaO_2,_ and PaCO_2_ at listing; pre-extubation PaO_2_, PaCO_2_ and PaO_2_/FiO_2_ratio; post-extubation PaO_2,_ PaCO_2_ and PaO_2_/FiO_2_ratio; Restrictive or Obstructive pattern at Pulmonary Function Tests; Post-operative Pulmonary Complications

BMI: body mass index, MELD: Model for End-stage Liver Disease, PaO_2_: partial pressure of arterial oxygen, PaCO_2_: partial pressure of arterial CO_2_, VVBP: Veno-Venous Bypass, D-MELD: Donor Model for End-stage Liver Disease, BAR: BAlance of Risk score, CIT: Cold Ischemia Time, ICU: Intensive Care Unit, SAPS: Simplified Acute Physiology Score, FiO_2_: Fraction of Inspired Oxygen, MEAF: Model for Early Allograft Function, RIFLE: Risk Injury Failure Loss End-stage of kidney disease

The competitive contribution of different factors to PRF was investigated. Non-collinear variables resulting more robustly associated with PFR were then combined in logistic analyses to quantify the impact of strongest factors in each category.

### Statistical analysis

Data were expressed as continuous or dichotomous variables. Continuous variables were reported as mean ± SD or as median and IQR. Dichotomous variables were reported as absolute (number) and relative (percentage) frequency. According to guidelines on statistical studies in organ transplantation [42], missing data relative to study covariates involved always less than 8.0% of cases. The incidence of PRF was compared between different patient groups using Chi squared test. Continuous variables were compared between patients with and without PRF using Student’s t‐test. Multivariate logistic regression analysis was performed to identify factors associated with PRF.

The relationship between factors and PRF was reported as Odd Ratio (OR) and 95% confidence intervals (CI). Due to the large number of potential factors, only those with p≤0.1 at univariate analysis were considered. According to the backward stepwise selection approach, variables with p>0.1 were eliminated. The goodness of fit of the final model was assessed using the Hosmer-Lemeshow test [43].

In order to avoid multicollinearity among similar parameters (for example, MELD vs MELDNa at the transplant) the potentially most performing parameters were identified using variance inflation factor statistics and ROC curve methodology [44]. Survival was expressed as patient survival and assessed by Kaplan Meier method and log-rank test. All analyses were performed with SPSS version 25.0 (Chicago, IL).

## Results

Overall, 212 consecutive transplants performed in 210 adult patients were identified. Twelve transplants were excluded (8 acute liver failure, 1 death at ICU arrival, 1 tracheotomy before LTx, and 2 early re-transplants due to primary non-function of the graft). In total, 200 transplants in 200 patients were studied. (S1 Fig). Among 200 patients, 7 had been previously admitted to the ICU (2 pneumonia, 2 variceal bleeding and 3 sepsis) and discharged before LTx. In contrast, 3 patients requiring hemofiltration were transplanted while staying in ICU. All patients arrived to ICU intubated and mechanically ventilated as per our protocol. Patients’ characteristics of the study population are summarized in Table 2.

**Table 2:** Characteristics of the study population and comparison between PRF and no-PRF cases (univariate analysis).

Factors	All (n = 200) Median (IQR/ Mean±SD/n (%)	*Missing* *n (%)*	PRF (n = 72) Median (IQR)/ Mean±SD/n (%)	no-PRF (n = 128) Median (IQR)/ Mean±SD/n (%)	*P value*
**PREOPERATIVE FACTORS (Recipient)**					
Age (years)	56 (48–62)	*0 (0)*	56 (48–62)	56 (48–62)	*0*.*90*
**Female sex**	**40 (20.0)**	*0 (0)*	**22 (30.6)**	**18 (14.1)**	***0*.*05***
BMI	25.6 ± 3.9	*0 (0)*	25.3 ± 3.9	25.8 ± 3.9	*0*.*37*
HCC	75 (37.5)	*0 (0)*	28 (38.9)	47 (36.7)	*0*.*76*
**MELD at LTx**	**18 (13–24)**	***0 (0)***	**22 (15–30)**	**17 (12–21)**	***0*.*05***
**MELD in HCC pts**	13 (11–18)	*0 (0)*	**15 (12–19)**	**12 (10–15)**	***0*.*02***
**MELD in no-HCC pts**	21 (16–27)	*0 (0)*	**25 (17–32)**	**20 (15–24)**	***<0*.*01***
**MELDNa at LTx**	**21 (15–28)**	*0 (0)*	**24 (16–32)**	**20 (14–25)**	***0*.*01***
MELDNa in HCC pts	16 (12–21)	*0 (0)*	19 (14–23)	15 (11–20)	*0*.*07*
**MELDNa in no-HCC pts**	**24 (19–30)**	*0 (0)*	**29 (22–33)**	**22 (18–27)**	***<0*.*01***
**Encephalopathy grade ≥2**	**7 (3.5)**	*0 (0)*	**4 (5.6)**	**3 (2.3)**	***<0*.*01***
**TLC** (% of predicted)	**91.4 ± 14.9**	*0(0)*	**88.0 ± 17.0**	**92.4 ± 13.6**	***0*.*02***
**FEV**_**1**_ (% of predicted)	**92.6 ± 17.3**	*0(0)*	**87.8 ± 19.5**	**95.3 ± 15.4**	***0*.*01***
**FVC** (% of predicted)	**96.9 ± 18.0**	*0 (0)*	**92.0 ± 20.5**	**99.9 ± 15.9**	***<0*.*01***
**Restrictive pattern**	**41 (20.5)**	***0 (0)***	**21 (29.2)**	**20 (15.6)**	***<0*.*01***
Obstructive pattern	14 (7.0)	***0 (0)***	6 (8.3)	8 (6.3)	*0*.*82*
**INTRAOPERATIVE FACTORS**					
**Portal vein thrombosis**	**15 (7.5)**	***5 (2)***	**9 (12.5)**	**6 (4.7)**	***0*.*04***
**VVBP**	**41 (20.5)**	***0 (0)***	**23 (31.9)**	**18 (14.1)**	***<0*.*01***
Porto-caval anastomosis	10 (5.0)	***0 (0)***	6 (8.3)	4 (3.1)	*0*.*11*
**Packed red blood cell (units)**	**10.6 ± 9.0**	***0 (0)***	**13.0 ± 10.0**	**9.1 ± 8.0**	***<0*.*01***
**Packed red blood cell >10 units**	**79 (39.5)**	***0 (0)***	**40 (55.6)**	**39 (30.5)**	***<0*.*01***
Fresh Frozen Plasma (units)	16.8 ± 16.6	***0 (0)***	18.8 ± 17.7	15.5 ± 15.7	*0*.*16*
**Platelets (units)**	**1.24 ± 1.4**	***0 (0)***	**1.69 ± 1.58**	**0.99 ± 1.20**	***<0*.*01***
**Operation time (hours)**	**12 (11–13)**	*3 (1)*	**12 (11–14)**	**11 (10–13)**	***0*.*03***
**LOGISTIC FACTORS**					
D-MELD at LTx	985 ± 529	*0 (0)*	1131 ± 586	903 ± 477	*0*.*46*
BAR	6.8 ± 4.3	*7 (3)*	8.1 ± 4.8	6.1 ± 3.7	*0*.*13*
**CIT (hours)**	**8 (7–8)**	***4 (2)***	**8 (7–9)**	**8 (7–8)**	***0*.*05***
**DONOR Factors**					
Age (years)	55 (38–67)	***0 (0)***	56 (43–68)	55 (38–66)	*0*.*95*
Non-standard donor	82 (41.0)	*16 (8)*	34 (47.2)	48 (37.5)	*0*.*21*
Extended criteria donor	94 (47.0)	*16 (8)*	37 (51.4)	57 (44.5)	*0*.*31*
**POST-OPERATIVE ICU factors**					
**SAPS II at ICU admission**	**36.1 ± 15.3**	*16 (8)*	**40.1 ± 15.6**	**33.8 ± 14.7**	***<0*.*01***
PaO_2_ pre-extubation (mmHg)	147.8 ± 35.8	*5 (2)*	144.2 ± 31.5	149.0 ± 39.0	*0*.*37*
**PaCO**_**2**_ **pre-extubation (mmHg)**	**36.5 ± 5.4**	***2 (1)***	**38.8 ± 5.6**	**34.5 ± 5.0**	***0*.*02***
PaO_2_/FiO_2_ pre-extubation	369 ± 104	*5 (2)*	352 ± 87	380± 111	*0*.*08*
**POST-OPERATIVE SURGICAL factors**					
**MEAF**	**4.9 ± 2.0**	*0 (0)*	**5.7 ± 2.0**	**4.5 ± 1.9**	***<0*.*01***
**MEAF 8 or higher**	**18 (9.0)**	*0 (0)*	**12 (16.7)**	**6 (4.7)**	***<0*.*01***
**MELD at the 3**^**rd**^**p.o.d.**	**15.9 ± 7.3**	***7 (3)***	**19.0 ± 7.0**	**14.2 ± 7.0**	***<0*.*01***
**Bilirubin at the 3**^**rd**^ **p.o.d(mg/dl)**	**5.2 ± 4.3**	*0 (0)*	**2.4 ± 0.8**	**1.8 ± 0.8**	***<0*.*01***
**RIFLE at the 3**^**rd**^**p.o.d.**	**0.43 ± 0.78**	*0 (0)*	**0.60 ± 0.90**	**0.33 ± 0.69**	***0*.*03***
**Creatinine at the 3**^**rd**^**p.o.d. (mg/dl)**	**1.29 ± 0.67**	*0 (0)*	**1.48 ± 0.68**	**1.18 ± 0.65**	***<0*.*01***
***OTHER DATA (available after 48 hours)***					
***PaO***_***2***_ ***post-extubation (mmHg)***	**115.8 ± 34.8**	***15 (8)***	**103.1 ± 33.9**	**123.4 ± 32.3**	***<0*.*01***
***PaCO***_***2***_ ***post-extubation (mmHg)***	**37.5 ± 6.0**	***15 (8)***	**39.2 ± 6.8**	**36.4 ± 5.1**	***<0*.*01***
***PaO***_***2***_***/FiO***_***2***_ ***post-extubation***	**283 ± 93**	***15 (8)***	**255 ± 100**	**300 ± 85**	***<0*.*01***
***Mechanical Ventilation (hours)***	**22 (17–44)**	*0 (0)*	**61 (35–91)**	**20 (16–25)**	***<0*.*01***
***Non-infectious lung involvement***	**113 (56.5)**	*0 (0)*	**53 (73.6)**	**60 (46.9)**	***<0*.*01***
***Pneumonia***	**22 (11.0)**	*0 (0)*	**20 (27.8)**	**2 (1.6)**	***<0*.*01***
***Clavien-Dindo stratification***					
***Grade 0***	**60 (30.0)**	*0 (0)*	**7 (9.7)**	**53 (41.4)**	***<0*.*01***
*Grade 1*	49 (24.5)	*0 (0)*	15 (20.8)	34 (26.6)	*0*.*36*
*Grade 2*	37 (18.5)	*0 (0)*	15 (20.8)	22 (17.2)	*0*.*52*
*Grade 3A*	13 (6.5)	*0 (0)*	7 (9.7)	6 (4.7)	*0*.*17*
*Grade 3B*	12 (6.0)	*0 (0)*	7 (9.7)	5 (3.9)	*0*.*05*
***Grade 4***	**15 (7.5)**	*0 (0)*	**9 (12.5)**	**6 (4.7)**	***0*.*04***
***Grade 5***	**14 (7.0)**	*0 (0)*	**12 (16.7)**	**2 (1.6)**	***<0*.*01***
***Grade 3B and higher***	**41 (20.5)**	*0 (0)*	**28 (38.9)**	**13 (10.2)**	***<0*.*01***

PRF: Postoperative Respiratory Failure, IQR: interquatile range, BMI: body mass index, LTx: Liver Transplantation, HCC: Hepatocellular carcinoma, MELD: Model for End-stage Liver Disease, LVEF%: Left Ventricular Ejection Fraction percentage, SPAP: Systolic Pulmonary Arterial Pressure, PPS: porto-pulmonary syndrome, PaO_2_: partial pressure of arterial oxygen, PaCO_2_: partial pressure of arterial CO_2_, TLC: Total Lung Capacity, FEV_1_: Forced Expiratory Flow in 1 second, FVC: Forced Vital Capacity, VVBP: Veno-Venous bypass, D-MELD: Donor Model for End-stage Liver Disease, BAR: BAlance of Risk score, CIT: Cold Ischemia Time, ICU: Intensive Care Unit, SAPS: Simplified Acute Physiology Score, FiO_2_: Fraction of Inspired Oxygen, MEAF: Model for Early Allograft Function, RIFLE: Risk Injury Failure Loss End-stage of kidney disease

### Predictors of PRF at univariate analysis

**Fig 1:**
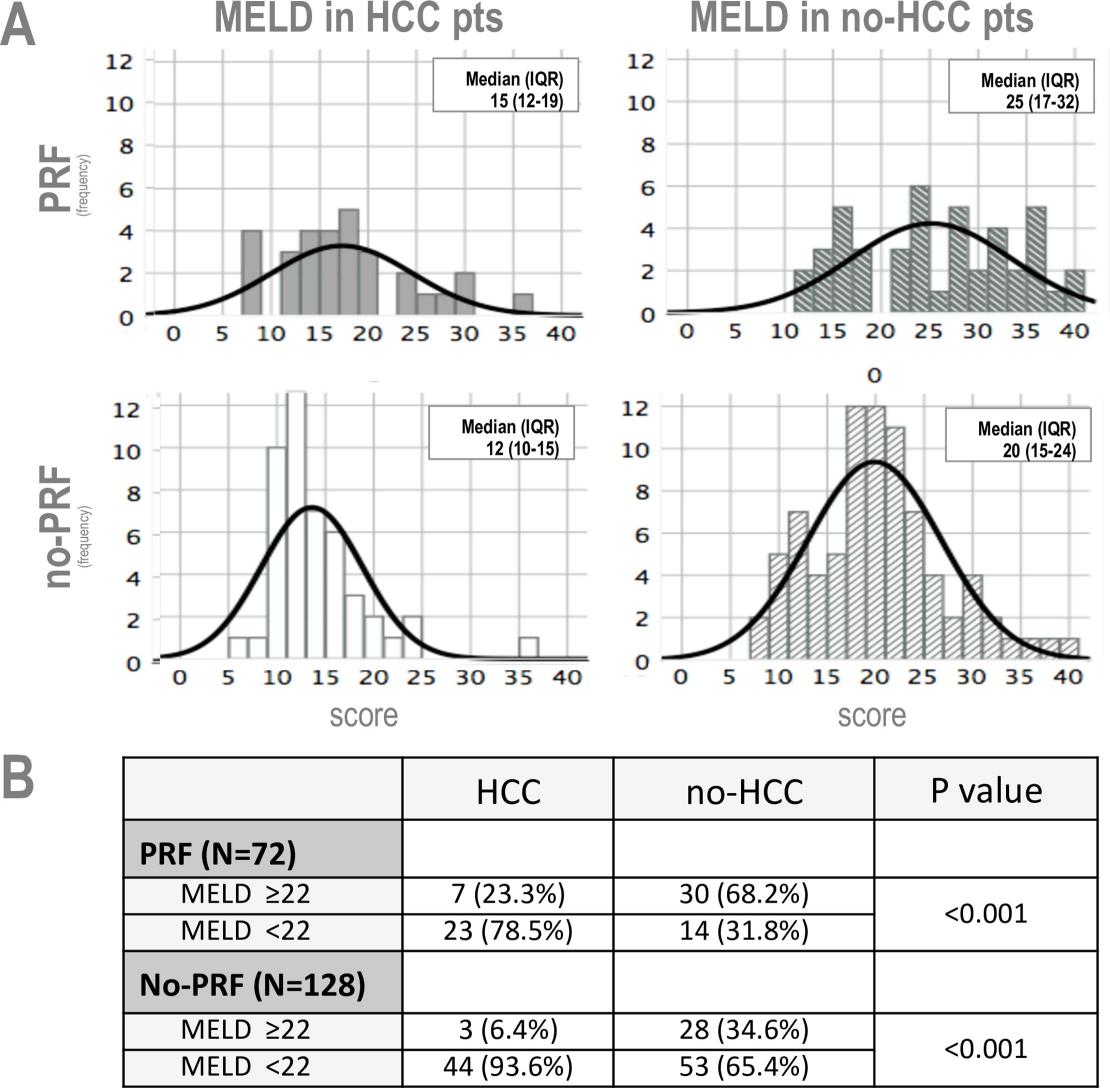
Frequencies of MELD in no-HCC and HCC patients. (A) Histograms of MELD according to the outcome (PRF vs no-PRF) are reported. For each subset mean ± SD and median (IQR) are reported. (B) Frequencies and (percentages) are reported in PRF and no-PRF patients according to MELD ≥22 and MELD <22.

Clinical characteristics of patients with and without PRF are summarized in Table 2 and S1 Table. PRF was observed in 72 (36.0%) out of 200 transplants. PRF and no-PRF cases had similar age, BMI and prevalence of diabetes and HCC. A higher proportion of cases in the PRF group were female, required pre-transplant or post-transplant hemofiltration, presented grade ≥2 encephalopathy or hepatopulmonary syndrome. Pulmonary function tests were altered in 55 out of 200 patients (restrictive pattern in 41 cases, 20.5% and obstructive pattern in 14 cases, 7.0%). Overall, the presence of restrictive pattern was significantly associated with PRF (Table 2). Among PRF patients with restrictive pattern, moderate-severe pleural effusion was observed in 35 cases (85.3%) and moderate-severe ascites was present in 12 cases (53.6%). On the whole, PRF cases exhibited higher values of MELD and MELDNa at transplant. In particular, MELD was higher in PRF patients independently from the HCC status, whereas, MELD in PRF patients with HCC was lower than in PRF without HCC (Fig 1).

PRF cases showed a higher incidence of portal thrombosis, received a higher number of packed red blood cell and platelet units, and more frequently required veno-venous bypass (VVBP). Furthermore, PRF patients displayed higher D-MELD, whilst Cold Ischemia Time (CIT) was moderately increased. The operation time in the PRF group was longer than in no-PRF group. Similarly, PRF cases had higher SAPS II scores at ICU admission. After surgery, PRF patients exhibited higher pre-extubation PaCO_2_ levels. Overall, 22 patients (11.0%) developed pneumonia, mostly in the PRF group (20, 27.8%). MEAF (evaluated as continuous variable and as percentage of patients with MEAF score >8), MELD at the 3^rd^ pod, bilirubin at the 3^rd^ pod, RIFLE and creatinine at the 3^rd^ pod were higher in PRF cases. Accordingly, PRF cases suffered a higher incidence of severe complications as assessed by the Clavien-Dindo stratification.

### Predictors of PRF at ROC and multivariate analysis

**Fig 2:**
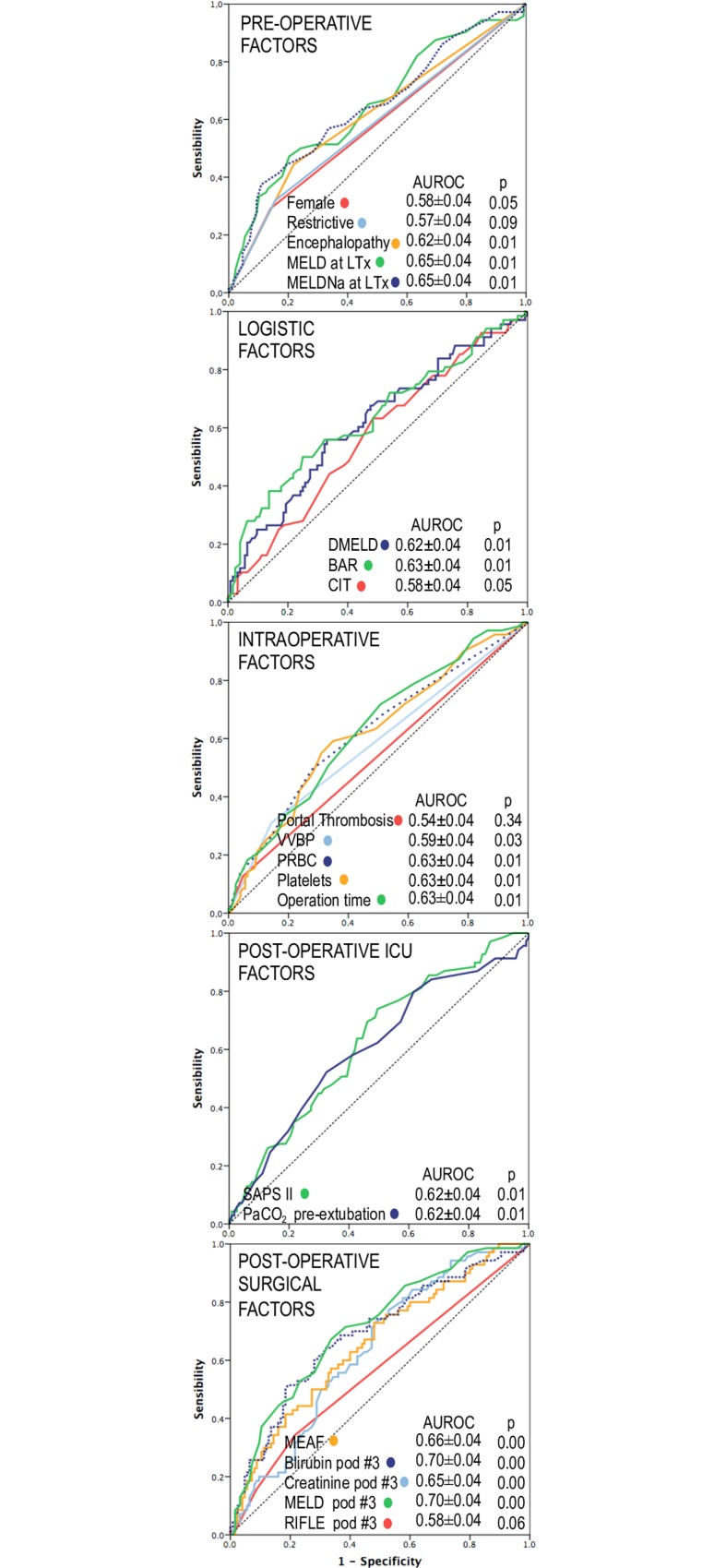
ROC curve analysis. The Areas Under the Curve and Standard Errors are reported under each subset.

Among the 28 PRF predictors at univariate analysis, hemofiltration and pre-LTx mechanical ventilation were not included for the exiguous number of positive cases. Six parameters were excluded because of collinearity. The remaining 22 parameters were investigated by ROC curve (Fig 2, S2 Table) and logistic regression analyses.

In addition, recipient age was included for epidemiologic reasons. Logistic regression analysis showed the following independent risk factors: recipient age (OR = 1.05; 95%CI 1.01–1.09; p = 0.010), female sex (OR = 2.79; 95%CI 1.19–6.52; p = 0.018), MELD at transplant (OR = 1.09; 95%CI 1.04–1.14; p<0.001), lung restrictive pattern (OR = 2.49; 95%CI 1.11–5.61; p = 0.027), VVBP (OR = 3.03; 95%CI 1.33–6.90; p = 0.008), pre-extubation PaCO_2_ (OR = 1.11; 95%CI 1.03–1.18; p = 0.003) and MEAF (OR = 1.37; 95%CI 1.15–1.63; p<0.001). Overall, 198 out of 200 observations with a full set of confounders were included in the multivariate analysis, The analysis displayed an excellent Hosmer-Lemeshow test value indicative of goodness of fit (p = 0.88). Detailed data are reported in S3 Table.

### Univariate analysis according to the type of PRF

Among PRF cases, 44 (61.1%) were attributable to WF and 28 (38.9%) to EF. Main causes of WF were hemodynamic instability, alveolar-interstitial pulmonary edema (frequently transfusion-associated circulatory overload in patients with massive bleeding), neurological impairment, and pending surgical issues (abdominal packing). In contrast, in EF cases, reinstitution of mechanical ventilation was mainly due to copious tracheal secretions with ineffective cough and atelectasis and concomitant respiratory muscle fatigue.

Demographic and clinical characteristics of EF and WF patients are shown in Table 3 and in S4 Table.

**Table 3:** Characteristics of PRF patients according to extubation and weaning failure (univariate analysis).

Factors	Extubation failure (n = 28) Median (IQR) / Mean ± SD/n (%)	Weaning failure (n = 44) Median (IQR) / Mean±SD /n (%)	*P value*
**PREOPERATIVE FACTORS (recipient)**			
Age (years)	58 (50–62)	54 (47–62)	*0*.*90*
Female sex	8 (28.6)	14 (31.8)	*0*.*77*
BMI >30	2 (7.1)	9 (20.5)	*0*.*13*
HCC	11 (39.3)	17 (38.6)	*0*.*96*
MELD at LTx	20 (14–26)	23 (15–31)	*0*.*06*
**MELDNa at LTx**	**23 (16–30)**	**27 (15–33)**	***0*.*01***
Encephalopathy grade ≥2	2 (7.1)	2 (4.5)	*0*.*22*
Restrictive pattern	7 (25.0)	14 (31.8)	*0*.*56*
Obstructive pattern	3 (10.7)	3 (6.8)	*0*.*62*
**INTRAOPERATIVE factors**			
Portal Vein thrombosis	2 (7.1)	7 (15.9)	*0*.*26*
**VVBP**	**5 (17.9)**	**18 (40.9)**	***0*.*04***
Packed red blood cell (units)	11.3 ± 9.9	14.1 ± 11.3	*0*.*24*
Packed red blood cell >10 units	12 (42.9)	28 (63.6)	*0*.*08*
Fresh Frozen Plasma (units)	14.5 ± 14.8	22.0 ± 19.1	*0*.*07*
Platelets (units)	1.60 ± 1.80	1.75 ± 1.43	*0*.*73*
Operation time (hours)	12 (12–14)	13 (12–14)	*0*.*58*
**LOGISTIC FACTORS**			
D-MELD at LTx	1067 ± 556	1172 ± 606	*0*.*56*
BAR	7.1 ± 4.0	8.8 ± 5.3	*0*.*12*
CIT (hours)	8 (7–8)	8 (7–9)	*0*.*23*
**POST-OPERATIVE ICU FACTORS**			
SAPS II at the ICU admission	37.8 ± 13.3	41.6 ± 16.9	*0*.*33*
PaCO_2_ pre-extubation (mmHg)	37.4 ± 5.6	38.0 ± 6.1	*0*.*68*
PaO_2_/FiO_2_ pre-extubation	372 ± 88	337 ± 85	*0*.*13*
**POST-OPERATIVE SURGICAL FACTORS**			
MEAF	5.3 ± 2.0	5.9 ± 1.9	*0*.*23*
MEAF 8 & over	3 (10.7)	9 (20.5)	*0*.*28*
MELD on 3^rd^ p.o.d. (mg/dl)	17.6 ± 6.2	18.9 ± 8.4	*0*.*17*
Bilirubin on 3^rd^ p.o.d. (mg/dl)	5.7 ± 4.3	8.0 ± 5.6	*0*.*06*
Creatinine on 3^rd^ p.o.d. (mg/dl)	1.32 ± 0.57	1.58 ± 0.73	*0*.*10*
***OTHER DATA (available after 48 hours)***			
***PaO***_***2***_ ***post-extubation (mmHg)***	**89.0 ± 32.0**	**113.9 ± 31.6**	***<0*.*01***
*PaCO*_*2*_ *post-extubation (mmHg)*	39.2 ± 7.8	39.3 ± 6.1	*0*.*96*
***PaO***_***2***_***/FiO***_***2***_ ***post-extubation***	**209 ± 93**	**290 ± 92**	***<0*.*01***
***Mechanical Ventilation (hours)***	**21 (14–39)**	**73 (66–119)**	***<0*.*01***
*Non-infectious lung involvement*	17 (60.7)	36 (81.8)	*0*.*22*
*Pneumonia*	7 (25.0)	13 (29.5)	*0*.*68*
***Clavien-Dindo Grade 3B and higher***	**5 (17.9)**	**23 (52.3)**	***<0*.*01***
*LoS in ICU post LTx (days)*	9 (7–15)	10 (7–18)	*0*.*81*
***Death in ICU***	**1 (3.6)**	**10 (22.7)**	***0*.*03***

PRF: Postoperative Respiratory Failure, BMI: body mass index, HCC: Hepatocellular carcinoma, MELD: Model for End-stage Liver Disease, LTx: Liver Transplantation, VVBP: Veno-Venous bypass, D-MELD: Donor Model for End-stage Liver Disease, BAR: BAlance of Risk score, CIT: Cold Ischemia Time, ICU: Intensive Care Unit, SAPS: Simplified Acute Physiology Score, PaO_2_: partial pressure of arterial oxygen, PaCO_2_: partial pressure of arterial CO_2_, FiO_2_: Fraction of Inspired Oxygen, MEAF: Model for Early Allograft Function, RIFLE: Risk Injury Failure Loss End-stage of kidney disease, LoS: Length of stay.

**Fig 3:**
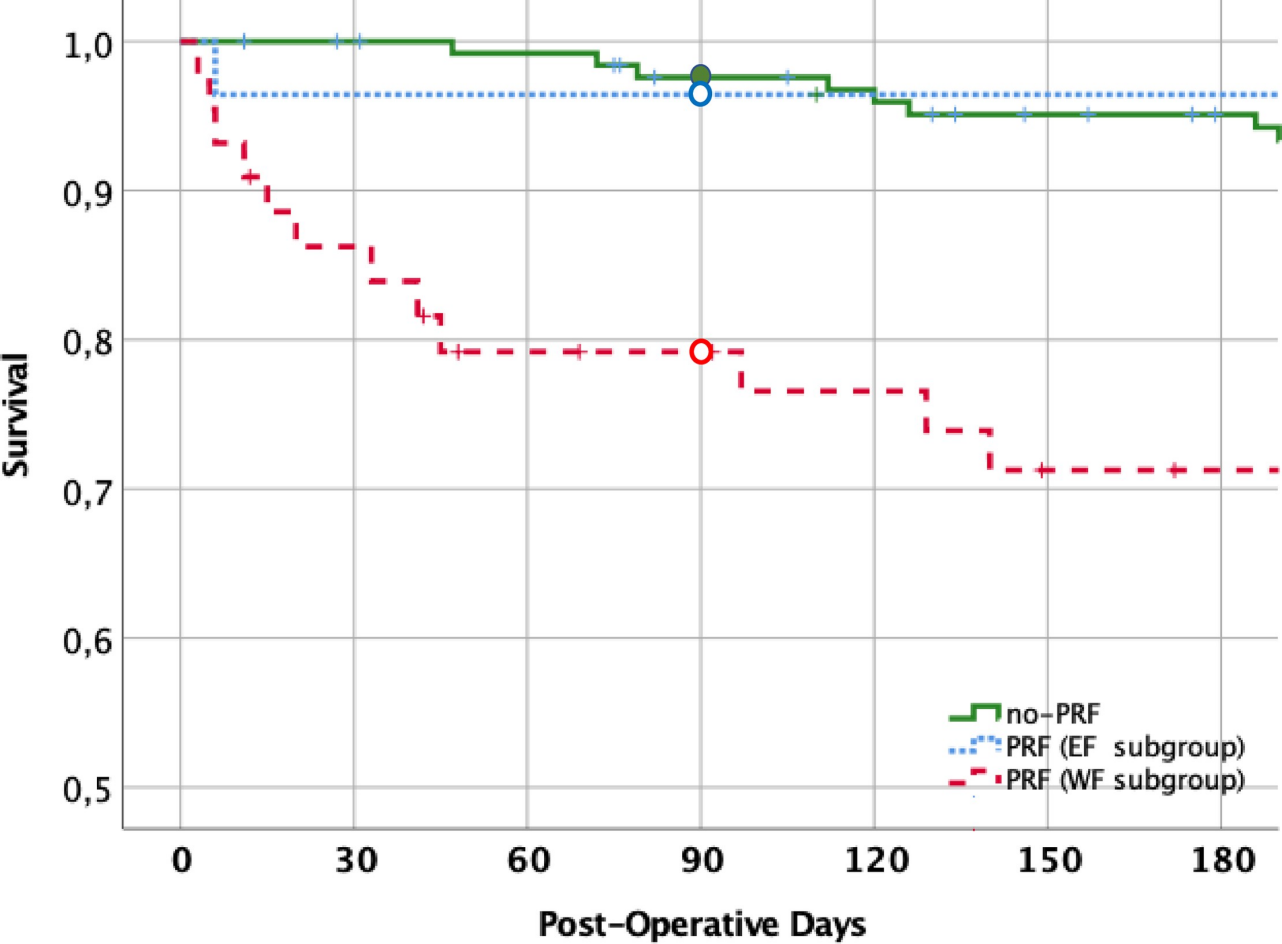
Survival analysis according with the PRF status. Patient survival at 90 days was 97.6%±1.4% in the no-PRF group (continuous line), 96.4%±3.5% in the EF subgroup (dash-interrupted line), and 79.2%±6.2% in the WF subgroup (dot-interrupted line). Survival was significantly different between PRF and no-PRF groups (p<0.001) and, within PRF patients, between EF and WF- subgroups (p = 0.047). WF, but not EF patient’ survival, differed from that of no-PRF patients.

On the whole, WF cases showed significantly higher values of MELDNa than EF patients, while MELD values were only slightly increased. Regarding intraoperative variables, WF cases showed a higher prevalence of VVBP (p = 0.04). Among additional postoperative data, EF cases displayed lower post-extubation PaO2/FiO2 ratio, lower rate of severe surgical complications, and lower ICU mortality rate (Fig 3).

### Length of stay, discharge and survival

Overall, LoS in ICU was 6 days (IQR 5–10 day). No-PRF cases had a median LoS of 5 days (IQR 4–7 day) and were all discharged from ICU. In contrast, PRF ones had a significantly higher LoS (10 days, IQR 7–18 day, p<0.001) in comparison with no-PRF patients. The LoS was similar in EF and WF groups (Table 3). The day-90 survival was 97.6%±1.4 in the no-PRF group (one patient died at the 80^th^ postoperative day for intractable ascites and sepsis) and 86.0%±4.1 in the entire PRF group (p<0.001). Among PRF patients, 90-day survival was 96.4%±3.5% and 79.2%±6.2% in the EF and WF subgroups, respectively (p = 0.047) (Fig 3).

## Discussion

The reported incidence of PRF in liver transplantation ranges between 11% and 42% due to the different thresholds used to define “prolonged” mechanical ventilation (from 24 hours to 7.5 days), and to the different inclusion criteria [11–18]. Overall, the incidence of PRF in our study population is 36.0%. We found that PRF is affected by seven independent variables, including MELD at transplant, restrictive lung pattern, use of VVBP, MEAF, pre-extubation PaCO_2_, patient age and sex. Remarkably, many variables considered in our analysis, such as intraoperative surgical factors (portal thrombosis and porto-caval anastomosis) or logistic risk factors (D-MELD, BAlance of Risk, CIT) have been never investigated, neither collinearity was ruled out in previous studies [6,11–18]. Furthermore, this is the first study including the MEAF in a multivariable prediction model for PRF.

Among the identified PRF risk factors, MELD at transplant, restrictive lung pattern and use of VVBP resulted the most relevant. The role of MELD [13,14,17] and restrictive lung pattern [16] has been previously evidenced. Notably, our study population includes two well-defined groups: high-MELD no-HCC patients and low-MELD HCC patients, and both groups have similar PRF prevalence. Regarding the restrictive lung pattern, it mainly resulted from pleural effusion (with or without ascites) causing basal atelectasis. Actually, while ascites is absent or drained in the early postoperative days, pleural effusion may even worsen due to the lung dysfunction and/or diaphragm trauma.

Regarding VVPB, in our experience it is used in case of high surgical complexity due to various conditions such as grade-III/IV portal thrombosis, Budd Chiari syndrome, huge liver in polycystic liver disease, late re-transplant. In addition, we occasionally used VVBP as a rescue procedure for massive bleeding, when the graft is not yet ready to be implanted (delay in the organ transport, complex reconstruction on the back table). The proportion of patients receiving VVBP in our study is comparable to that previously reported [16]. Moreover, in our series of patients, it can be considered as a surrogate marker of portal hypertension/thrombosis, it is not collinear with MELD, neither it was routinely used in our high-MELD cases. VVBP prolongs *per se* the operation time, activates fibrinolysis and platelets’ consumption [45]. Although the operation time showed the highest area under the curve at ROC analysis, VVBP was more predictive in all decile categories at the Hosmer-Lemeshow test. We are aware that in centers not using VVPB, other indicators might be predictive for surgical complexity.

In our population, PRF risk markedly increased along the MEAF score. The association between graft recovery and PRF has been scarcely investigated. High transaminases have been associated with PRF [11,18] or PaO_2_/FiO_2_ ≤300 mmHg [19]. This is the first study investigating the PRF impact of the MEAF score, obtained by bilirubin, transaminase and INR, and ranging continuously from 1 to 10 [29]. More recently, a novel score predicting graft recovery has been developed, based on bilirubin, transaminase, INR and platelet count [46]. Although more performant than MEAF, it includes parameters gathered until pod 10, and it is not suitable to evaluate PRF at 48 hours.

The mean value of pre-extubation PaCO_2,_ even though still normal, was higher in PRF patients. This finding has been previously reported [6] and it is conceivable that it results from a problematic weaning: notwithstanding the exact underlying mechanisms is presently unknown, the contribution of graft dysfunction/non-function or phrenic and/or diaphragmatic surgical trauma cannot be excluded.

As previously reported, we observed a negative prognostic effect for recipient age and female sex at multivariate analysis [17,47]. It has been suggested that females are less likely to receive intraoperative low-tidal protective ventilation during surgery, and this may yield a higher rate of postoperative respiratory complications [47]. Moreover, as compared to male patients, MELD score in females underestimates liver-kidney dysfunction up to 3 points, probably for the lower dietary intake and the lower tubular secretion of creatinine [48].

Of note, we failed to demonstrate any impact on the PRF occurrence of diabetes, BMI and, for the exiguous number of cases, of re-transplant [11–18,49,50].

Noteworthy, we observed that PRF, as usually defined [3,16], includes two populations of patients with different 90-day survival. Notably, the EF patients exhibited similar overall survival as no-PRF patients, whereas they did not differ from WF patients regarding main baseline clinical characteristics and PRF prognostic factors. It could be argued that these patients may have been extubated a quite bit early, as suggested by their lower post-extubation PaO_2_/FiO_2_. The assessment for readiness for extubation is based on several static measures, even though the dynamic nature of the weaning makes difficult to definitely predict its success [23]. Indeed, the EF occurs more frequently in patients unable to manage copious tracheal secretion due to ineffective cough, or respiratory fatigue secondary to poor patient cooperation or malnutrition. However, all these issues cannot be anticipated before extubation. Accordingly, EF patients compared with WF patients for LoS in ICU, whilst they had a significantly lower 90-day survival.

Although these observations have been gathered in a small subset of patients, we can hypothesize that EF group represents a low-risk subset of LTx patients, with lower prevalence of severe complications (Clavien-Dindo grade 3B and over), resulting in better survival. Overall, these findings show that differentiating among PRF patients those with EF from those with WF, can have a significant prognostic relevance.

Our study suffers from some limitations. It is based on a single-center experience, and has a retrospective design, whereas, to the best of our knowledge, PRF has not been investigated in multi-center prospective cohorts. According to previous studies, the role of PRF was investigated at 48 hours categorizing PRF as a dichotomic variable.[3–4] Nevertheless, our findings pave the way to future studies exploring the prognostic role of PRF as a time-dependent s variable. In our series of patients, functional capacity, frailty and sarcopenia were not prospectively recorded and were not included in the analyses, whereas BMI, a surrogate method to evaluate sarcopenia, was not predictive. The Dindo-Clavien grading of complications was used instead of the new Comprehensive Complication Index [51]. Furthermore, we did not test the Donor Risk Index [10], which is not applicable in Italy for the shorter distance between donor and recipient hospitals and higher donor age [52]. Finally, the present study does not include pediatric patients, adult patients transplanted for acute liver failure and living-donor transplants.

The multidisciplinary approach to predict PRF in the complex context of LTx deserves consideration. The methodology adopted may provide hepatologists and surgeons an adequate tool to estimate the ICU prognosis of listed patients. Patients with high MELD, restrictive lung pattern and/or high surgical complexity should be informed on their high PRF risk, eventually increased by early allograft dysfunction. Likewise, our study may help surgeons and intensivists to identify risk-mitigation strategies in specific patients, such as a wider use of non-invasive ventilation soon after the extubation. Accordingly, the frequent use of non-invasive ventilation, avoiding endotracheal reintubation, can explain the lower pulmonary infection rate of our population in comparison with other studies [6,16]. In fact, non-invasive ventilation prevents basal atelectasis due to abdominal distension, alteration of diaphragmatic function and contractility, promotes lung recruitment and decreases work of breathing [24].

## Conclusions

On the whole, our analysis carried out according to the organ-based perspective, lung restrictive pattern, native liver (MELD), surgical complexity, as captured by VVBP, and new liver function (MEAF) are the main determinants for PRF in non-acute LTx patients. Remarkably, donor variables, as evaluated before the transplant, do not always reflect the postoperative donor-related risk, which can be actually established only in the early postoperative days.

## Supporting information

S1 Fig Consort diagram. Eligibility, exclusion and study population
(TIF)

S1 Table Characteristics of the study population and comparison between PRF and no-PRF cases at univariate analysis. PRF: Postoperative Respiratory Failure, IQR: interquatile range, BMI: body mass index, LTx: Liver Transplantation, HCC: Hepatocellular carcinoma, MELD: Model for End-stage Liver Disease, LVEF%: Left Ventricular Ejection Fraction percentage, SPAP: Systolic Pulmonary Arterial Pressure, PPS: porto-pulmonary syndrome, PaO_2_: partial pressure of arterial oxygen, PaCO_2_: partial pressure of arterial CO_2_, TLC: Total Lung Capacity, FEV_1_: Forced Expiratory Flow in 1 second, FVC: Forced Vital Capacity, VVBP: Veno-Venous bypass, D-MELD: Donor Model for End-stage Liver Disease, BAR: BAlance of Risk score, CIT: Cold Ischemia Time, ICU: Intensive Care Unit, SAPS: Simplified Acute Physiology Score, FiO_2_: Fraction of Inspired Oxygen, MEAF: Model for Early Allograft Function, RIFLE: Risk Injury Failure Loss End-stage of kidney disease.
(DOCX)

S2 Table List of variables investigated by ROC curve analysis. (DOCX)

S3 Table Details of the multivariate analysis (logistic regression). The number of events (70) allowed the identification of 7 predictive factors. Hosmer-Lemeshow test: Chi^2^ = 3.78, p = 0.88). Age, MELD at transplant and pre-extubation PaCO_2_ showed a broad IQR (Interquartile Range) excursion, explaining how the low OR (Odd Ratio) is indicative of a strong statistical effect. MELD: Model for End-stage Liver Disease, VVBP: Veno-Venous bypass, PaCO_2_: partial pressure of arterial CO_2_, MEAF: Model for Early Allograft Function.
(DOCX)

S4 Table Characteristics of PRF patients according to extubation and weaning failure (univariate analysis). PRF: Postoperative Respiratory Failure, BMI: body mass index, HCC: Hepatocellular carcinoma, MELD: Model for End-stage Liver Disease, LTx: Liver Transplantation, VVBP: Veno-Venous bypass, D-MELD: Donor Model for End-stage Liver Disease, BAR: BAlance of Risk score, CIT: Cold Ischemia Time, ICU: Intensive Care Unit, SAPS: Simplified Acute Physiology Score, PaO_2_: partial pressure of arterial oxygen, PaCO_2_: partial pressure of arterial CO_2_, FiO_2_: Fraction of Inspired Oxygen, MEAF: Model for Early Allograft Function, RIFLE: Risk Injury Failure Loss End-stage of kidney disease, LoS: Length of stay.
(DOCX)

## Abbreviations

BMI: body mass index
CI: confidence intervals
CIT: Cold Ischemia Time
D-MELD: Donor Model for End-stage Liver Disease
EF: Extubation Failure
FiO_2_: Fraction of Inspired Oxygen
HCC: Hepatocellular Carcinoma
ICU: Intensive Care Unit
IQR: Interquartile Range
LoS: Length of Stay
LTx: Liver Transplantation
MEAF: Model for Early Allograft Function
MELD: Model for End-Stage Liver Disease
MELDNa: Model for End-Stage Liver Disease Sodium
OR: Odd Ratio
PaCO_2_: partial pressure of arterial CO_2_
PaO_2_: partial pressure of arterial oxygen
pod: postoperative day
PRF: Postoperative Respiratory Failure
RIFLE: Risk Injury Failure Loss End-stage of kidney disease
ROC: Receiver Operator Characteristic
SAPS: Simplified Acute Physiology Score
SD: Standard Deviation
VVBP: veno-venous bypass
WF: Weaning Failure
