# Supplementary figures and images for: Postoperative respiratory failure in liver transplantation: Risk factors and effect on prognosis
Source: PLoS One. 2019 Feb 11;14(2):e0211678. doi: 10.1371/journal.pone.0211678 (PMC6370207; doi:10.1371/journal.pone.0211678)

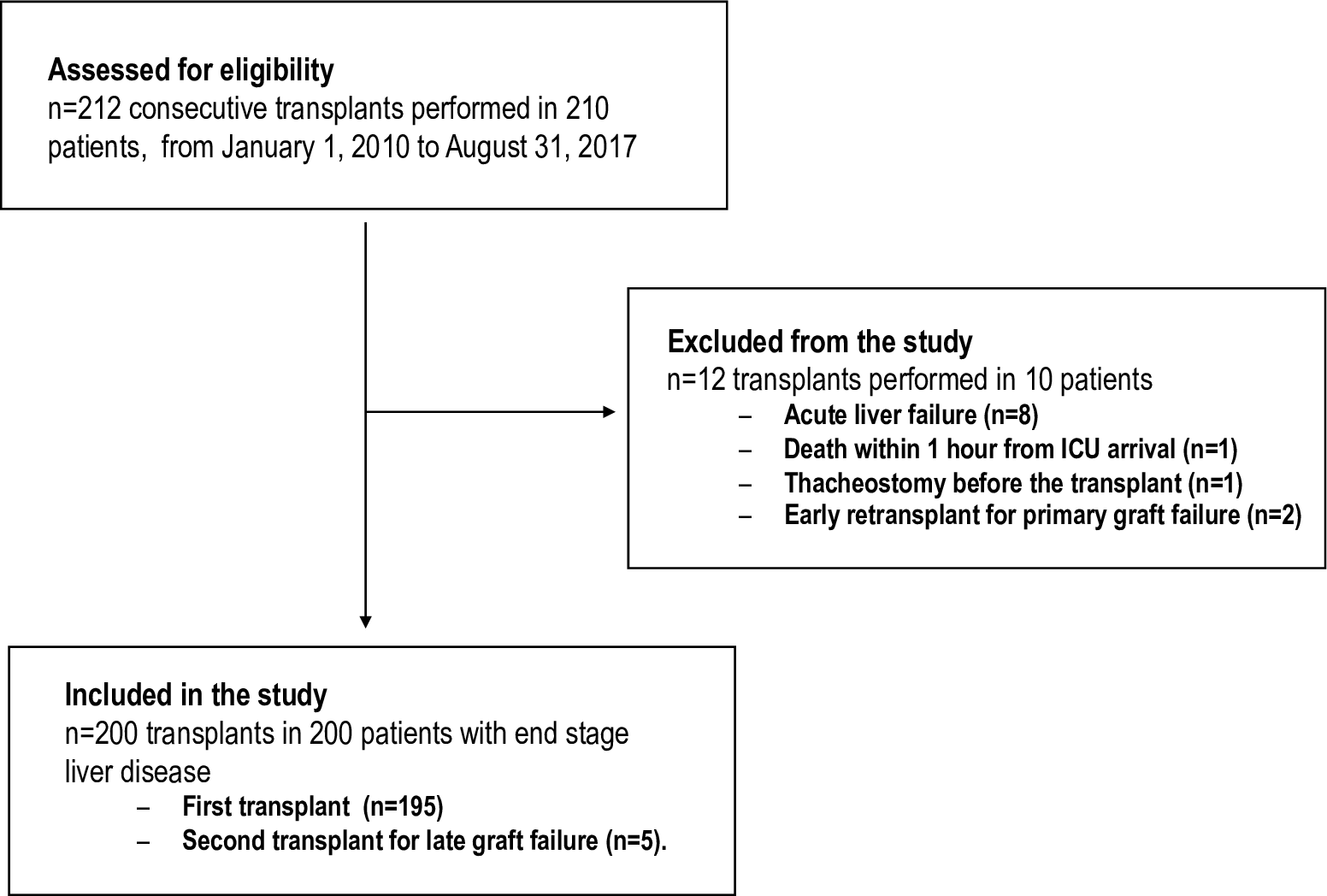

Supplement: S1 Fig — Eligibility, exclusion and study population (TIF) [file pone.0211678.s001.tif]
