# Supplementary material for: Postoperative respiratory failure in liver transplantation: Risk factors and effect on prognosis
Source: PLoS One. 2019 Feb 11;14(2):e0211678. doi: 10.1371/journal.pone.0211678 (PMC6370207; doi:10.1371/journal.pone.0211678)
Supplement: S1 Table — PRF: Postoperative Respiratory Failure, IQR: interquatile range, BMI: body mass index, LTx: Liver Transplantation, HCC: Hepatocellular carcinoma, MELD: Model for End-stage Liver Disease, LVEF%: Left Ventricular Ejection Fraction percentage, SPAP: Systolic Pulmonary Arterial Pressure, PPS: porto-pulmonary syndrome, PaO2: partial pressure of arterial oxygen, PaCO2: partial pressure of arterial CO2, TLC: Total Lung Capacity, FEV1: Forced Expiratory Flow in 1 second, FVC: Forced Vital Capacity, VVBP: Veno-Venous bypass, D-MELD: Donor Model for End-stage Liver Disease, BAR: BAlance of Risk score, CIT: Cold Ischemia Time, ICU: Intensive Care Unit, SAPS: Simplified Acute Physiology Score, FiO2: Fraction of Inspired Oxygen, MEAF: Model for Early Allograft Function, RIFLE: Risk Injury Failure Loss End-stage of kidney disease. (DOCX) [file pone.0211678.s002.docx]

|  | | | | | |
| --- | --- | --- | --- | --- | --- |
| **Factors** | **All (n=200)**  **Median (IQR/ Mean±SD/n (%)** | ***Missing***  ***n (%)*** | **PRF (n=72)**  **Median (IQR)/ Mean±SD/n (%)** | **no-PRF (n=128)**  **Median (IQR)/ Mean±SD/n (%)** | ***P value*** |
| **PREOPERATIVE FACTORS (Recipient)** | | | | |  |
| Age (years) | 56 (48-62) | *0 (0)* | 56 (48-62) | 56 (48-62) | *0.90* |
| **Female sex** | **40 (20.0)** | *0 (0)* | **22 (30.6)** | **18 (14.1)** | ***0.05*** |
| BMI | 25.6 ± 3.9 | *0 (0)* | 25.3 ± 3.9 | 25.8 ± 3.9 | *0.37* |
| BMI ≤18 | 6 (3.0) | *0 (0)* | 4 (5.6) | 2 (1.6) | *0.11* |
| BMI >30 | 28 (14.0) | *0 (0)* | 11 (15.3) | 17 (13.2) | *0.69* |
| HCC | 75 (37.5) | *0 (0)* | 28 (38.9) | 47 (36.7) | *0.76* |
| **MELD at LTx** | **18 (13-24)** | ***0 (0)*** | **22 (15-30)** | **17 (12-21)** | ***0.05*** |
| **MELD in HCC pts** | 13 (11-18) | *0 (0)* | **15 (12-19)** | **12 (10-15)** | ***0.02*** |
| **MELD in non-HCC pts** | 21 (16-27) | *0 (0)* | **25 (17-32)** | **20 (15-24)** | ***<0.01*** |
| **MELDNa at LTx** | **21 (15-28)** | *0 (0)* | **24 (16-32)** | **20 (14-25)** | ***0.01*** |
| MELDNa in HCC pts | 16 (12-21) | *0 (0)* | 19 (14-23) | 15 (11-20) | *0.07* |
| **MELDNa in non-HCC pts** | **24 (19-30)** | *0 (0)* | **29 (22-33)** | **22 (18-27)** | ***<0.01*** |
| **Encephalopathy grade ≥2** | **7 (3.5)** | *0 (0)* | **4 (5.6)** | **3 (2.3)** | ***<0.01*** |
| Diabetes | 44 (22.0) | ***2 (1)*** | 17 (23.6) | 27 (21.1) | *0.68* |
| LVEF % at echocardiography | 64.0 ± 5.0 | ***15 (8)*** | 63.6 ± 4.9 | 64.1 ± 5.0 | *0.53* |
| LVEF % <50% | 1 (0.5) | ***16 (8)*** | 0 (0.0) | 1 (0.8) | *0.45* |
| SPAP at echocardiography (mmHg) | 28.3 ± 6.4 | ***15 (8)*** | 29.4 ± 6.5 | 27.6 ± 6.3 | *0.11* |
| Diastolic dysfunction (moderate) | 14 (7.0) | *16 (0)* | 5 (6.9) | 9 (7.0) | *0.97* |
| PPS (PaO_2_≥45 mmHg) | 4 (2.0) | *0 (0)* | 1 (1.4) | 3 (2.3) | *0.63* |
| PaO_2_ in room air at listing (mmHg) | 88.2 ± 14.0 | *16 (8)* | 87.5 ± 14.8 | 89.5 ± 13.4 | *0.09* |
| PaCO_2_ at listing (mmHg) | 32.1 ± 4.3 | *16 (8)* | 32.1 ± 4.9 | 32.1 ± 3.9 | *0.98* |
| pH at listing | 7.44 ± 0.03 | *16 (8)* | 7.44 ± 0.04 | 7.44± 0.03 | *0.94* |
| **TLC** (% of predicted**)** | **91.4 ± 14.9** | *0(0)* | **88.0 ± 17.0** | **92.4 ± 13.6** | ***0.02*** |
| **FEV_1_** (% of predicted**)** | **92.6 ± 17.3** | *0(0)* | **87.8 ± 19.5** | **95.3 ± 15.4** | ***0.01*** |
| **FVC** (% of predicted**)** | **96.9 ± 18.0** | *0 (0)* | **92.0 ± 20.5** | **99.9 ± 15.9** | ***<0.01*** |
| FEV_1_/FVC (% of predicted) | 76.8 ± 10.4 | *0(0)* | 79.3 ± 7.3 | 77.8 ± 6.1 | *0.41* |
| **Restrictive pattern** | **41 (20.5)** | ***0 (0)*** | **21 (29.2)** | **20 (15.6)** | ***<0.01*** |
| Obstructive pattern | 14 (7.0) | ***0 (0)*** | 6 (8.3) | 8 (6.3) | *0.82* |
| **INTRAOPERATIVE FACTORS** | | | | | |
| **Portal vein thrombosis** | **15 (7.5)** | ***5 (2)*** | **9 (12.5)** | **6 (4.7)** | ***0.04*** |
| Previous abdominal surgery | 29 (14.5) | ***2 (1)*** | 11 (15.3) | 18 (14.1) | *0.80* |
| **VVBP** | **41 (20.5)** | ***0 (0)*** | **23 (31.9)** | **18 (14.1)** | ***<0.01*** |
| Porto-caval anastomosis | 10 (5.0) | ***0 (0)*** | 6 (8.3) | 4 (3.1) | *0.11* |
| **Packed red blood cell (units)** | **10.6 ± 9.0** | ***0 (0)*** | **13.0 ± 10.0** | **9.1 ± 8.0** | ***<0.01*** |
| **Packed red blood cell >10 units** | **79 (39.5)** | ***0 (0)*** | **40 (55.6)** | **39 (30.5)** | ***<0.01*** |
| Fresh Frozen Plasma (units) | 16.8 ± 16.6 | ***0 (0)*** | 18.8 ± 17.7 | 15.5 ± 15.7 | *0.16* |
| **Platelets (units)** | **1.24 ± 1.4** | ***0 (0)*** | **1.69 ± 1.58** | **0.99 ± 1.20** | ***<0.01*** |
| **Operation time (hours)** | **12 (11-13)** | *3 (1)* | **12 (11-14)** | **11 (10-13)** | ***0.03*** |
| **LOGISTIC FACTORS** | | | | | |
| D-MELD at LTx | 985 ± 529 | *0 (0)* | 1131 ± 586 | 903 ± 477 | *0.46* |
| BAR | 6.8 ± 4.3 | *7 (3)* | 8.1 ± 4.8 | 6.1 ± 3.7 | *0.13* |
| **CIT (hours)** | **8 (7-8)** | ***4 (2)*** | **8 (7-9)** | **8 (7-8)** | ***0.05*** |
| **DONOR Factors** | | | | | |
| Age (years) | 55 (38-67) | ***0 (0)*** | 56 (43-68) | 55 (38-66) | *0.95* |
| Non-standard donor | 82 (41.0) | *16 (8)* | 34 (47.2) | 48 (37.5) | *0.21* |
| Extended criteria donor | 94 (47.0) | *16 (8)* | 37 (51.4) | 57 (44.5) | *0.31* |
| **POST-OPERATIVE ICU factors** | | | | | |
| **SAPS II at ICU admission** | **36.1 ± 15.3** | *16 (8)* | **40.1 ± 15.6** | **33.8 ± 14.7** | ***<0.01*** |
| PaO_2_ pre-extubation (mmHg) | 147.8 ± 35.8 | *5 (2)* | 144.2 ± 31.5 | 149.0 ± 39.0 | *0.37* |
| **PaCO_2_ pre-extubation (mmHg)** | **36.5 ± 5.4** | ***2 (1)*** | **38.8 ± 5.6** | **34.5 ± 5.0** | ***0.02*** |
| PaO_2_/FiO_2_ pre-extubation | 369 ± 104 | *5 (2)* | 352 ± 87 | 380± 111 | *0.08* |
| **POST-OPERATIVE SURGICAL factors** | | | | | |
| **MEAF** | **4.9 ± 2.0** | *0 (0)* | **5.7 ± 2.0** | **4.5 ± 1.9** | ***<0.01*** |
| **MEAF 8 or higher** | **18 (9.0)** | *0 (0)* | **12 (16.7)** | **6 (4.7)** | ***<0.01*** |
| **MELD at the 3^rd^p.o.d.** | **15.9 ± 7.3** | ***7 (3)*** | **19.0 ± 7.0** | **14.2 ± 7.0** | ***<0.01*** |
| **Bilirubin at the 3^rd^ p.o.d(mg/dl)** | **5.2 ± 4.3** | *0 (0)* | **2.4 ± 0.8** | **1.8 ± 0.8** | ***<0.01*** |
| **RIFLE at the 3^rd^p.o.d.** | **0.43 ± 0.78** | *0 (0)* | **0.60 ± 0.90** | **0.33 ± 0.69** | ***0.03*** |
| **Creatinine at the 3^rd^p.o.d. (mg/dl)** | **1.29 ± 0.67** | *0 (0)* | **1.48 ± 0.68** | **1.18 ± 0.65** | ***<0.01*** |
| ***OTHER DATA (available after 48 hours)*** | | | | | |
| ***PaO_2_ post-extubation (mmHg)*** | **115.8 ± 34.8** | ***15 (8)*** | **103.1 ± 33.9** | **123.4 ± 32.3** | ***<0.01*** |
| ***PaCO_2_ post-extubation (mmHg)*** | **37.5 ± 6.0** | ***15 (8)*** | **39.2 ± 6.8** | **36.4 ± 5.1** | ***<0.01*** |
| ***PaO_2_/FiO_2_ post-extubation*** | **283 ± 93** | ***15 (8)*** | **255 ± 100** | **300 ± 85** | ***<0.01*** |
| ***Mechanical Ventilation (hours)*** | **22 (17-44)** | *0 (0)* | **61 (35-91)** | **20 (16-25)** | ***<0.01*** |
| ***Non-infectious lung involvement*** | **113 (56.5)** | *0 (0)* | **53 (73.6)** | **60 (46.9)** | ***<0.01*** |
| ***Pneumonia*** | **22 (11.0)** | *0 (0)* | **20 (27.8)** | **2 (1.6)** | ***<0.01*** |
| ***Clavien-Dindo stratification*** |  |  |  |  |  |
| ***Grade 0*** | **60 (30.0)** | *0 (0)* | **7 (9.7)** | **53 (41.4)** | ***<0.01*** |
| *Grade 1* | 49 (24.5) | *0 (0)* | 15 (20.8) | 34 (26.6) | *0.36* |
| *Grade 2* | 37 (18.5) | *0 (0)* | 15 (20.8) | 22 (17.2) | *0.52* |
| *Grade 3A* | 13 (6.5) | *0 (0)* | 7 (9.7) | 6 (4.7) | *0.17* |
| *Grade 3B* | 12 (6.0) | *0 (0)* | 7 (9.7) | 5 (3.9) | *0.05* |
| ***Grade 4*** | **15 (7.5)** | *0 (0)* | **9 (12.5)** | **6 (4.7)** | ***0.04*** |
| ***Grade 5*** | **14 (7.0)** | *0 (0)* | **12 (16.7)** | **2 (1.6)** | ***<0.01*** |
| ***Grade 3B and higher*** | **41 (20.5)** | *0 (0)* | **28 (38.9)** | **13 (10.2)** | ***<0.01*** |

**S1 Table. Characteristics of the study population and comparison between PRF and no-PRF cases at univariate analysis**

PRF: Postoperative Respiratory Failure, IQR: interquatile range, BMI: body mass index, LTx: Liver Transplantation, HCC: Hepatocellular carcinoma, MELD: Model for End-stage Liver Disease, LVEF%: Left Ventricular Ejection Fraction percentage, SPAP: Systolic Pulmonary Arterial Pressure, PPS: porto-pulmonary syndrome, PaO_2_: partial pressure of arterial oxygen, PaCO_2_: partial pressure of arterial CO_2_, TLC: Total Lung Capacity, FEV_1_: Forced Expiratory Flow in 1 second, FVC: Forced Vital Capacity, VVBP: Veno-Venous bypass, D-MELD: Donor Model for End-stage Liver Disease, BAR: BAlance of Risk score, CIT: Cold Ischemia Time, ICU: Intensive Care Unit, SAPS: Simplified Acute Physiology Score, FiO_2_: Fraction of Inspired Oxygen, MEAF: Model for Early Allograft Function, RIFLE: Risk Injury Failure Loss End-stage of kidney disease.
