# Supplementary material for: Postoperative respiratory failure in liver transplantation: Risk factors and effect on prognosis
Source: PLoS One. 2019 Feb 11;14(2):e0211678. doi: 10.1371/journal.pone.0211678 (PMC6370207; doi:10.1371/journal.pone.0211678)
Supplement: S2 Table — (DOCX) [file pone.0211678.s003.docx]

**S2 Table**. **List of variables investigated by ROC curve analysis.**

|  | | | |
| --- | --- | --- | --- |
| **Factors** | **AUROC ± SE** | ***P* value** | **95% CI** |
| **PREOPERATIVE FACTORS (recipient)** | | | |
| Age (years) | 0.51± 0.04 | 0.95 | 0.43 - 0.59 |
| **Female sex** | **0.58± 0.04** | **0.05** | **0.45 - 0.67** |
| Restrictive/non-restrictive pattern | 0.57± 0.04 | 0.09 | 0.49 - 0.66 |
| Endotracheal intubation before LTx | 0.51 ± 0.04 | 0.71 | 0.42 - 0.60 |
| ***Hepatic encephalopathy ≥2*** | ***0.62± 0.04*** | ***0.01*** | ***0.53 – 0.70*** |
| ***MELD at LTx*** | ***0.65± 0.04*** | ***0.01*** | ***0.56- 0.73*** |
| ***MELDNa at LTx*** | ***0.65± 0.04*** | ***0.01*** | ***0.56- 0.73*** |
| **LOGISTIC FACTORS** | | | |
| ***D-MELD at LTx*** | ***0.62± 0.04*** | ***0.01*** | ***0.54 - 0.70*** |
| ***BAR*** | ***0.63± 0.04*** | ***0.01*** | ***0.54- 0.71*** |
| **CIT (hours)** | **0.58± 0.04** | **0.05** | **0.50 - 0.67** |
| **INTRAOPERATIVE FACTORS** | | | |
| *Portal Vein Thrombosis* | *0.54 ± 0.04* | *0.34* | *0.45– 0.63* |
| ***VVBP*** | ***0.59± 0.04*** | ***0.03*** | ***0.51 - 0-67*** |
| ***Packed Red Blood Cell (units)*** | ***0.63 ± 0.04*** | ***0.01*** | ***0.54- 0.71*** |
| ***Platelets (units)*** | ***0.63 ± 0.04*** | ***0.01*** | ***0.55- 0.71*** |
| ***Operation time (hours)*** | ***0.63 ± 0.04*** | ***0.01*** | ***0.55 - 0.71*** |
| **POST-OPERATIVE ICU FACTORS** |  |  |  |
| ***SAPS II*** | ***0.62 ± 0.04*** | ***0.01*** | ***0.55 - 0.70*** |
| ***PaCO_2_pre-extubation (mmHg)*** | ***0.62 ± 0.04*** | ***0.01*** | ***0.54 - 0.70*** |
| **POST-OPERATIVE SURGICAL FACTORS** | | | |
| ***MEAF*** | ***0.66 ± 0.04*** | ***<0.01*** | ***0.58 - 0.74*** |
| ***Bilirubin at the 3^rd^p.o.d.*** | ***0.70 ± 0.04*** | ***<0.01*** | ***0.63 - 0.77*** |
| ***Creatinine at the 3^rd^p.o.d.*** | ***0.65 ± 0.04*** | ***<0.01*** | ***0.57 - 0.73*** |
| ***MELD at the 3^rd^p.o.d.*** | ***0.70 ± 0.04*** | ***<0.01*** | ***0.63 - 0.77*** |
| RIFLE at the 3^rd^p.o.d. | 0.58 ± 0.04 | 0.06 | 0.50 - 0.66 |
| ROC: Receiver Operator Characteristic, AUROC: Area Under ROC curve, SE: Standard Error, CI: Confidence Interval, LTx: Liver Transplantation, MELD: Model for End-stage Liver Disease, D-MELD: Donor Model for End-stage Liver Disease, BAR: BAlance of Risk score, CIT: Cold Ischemia Time, VVBP: Veno-Venous bypass, ICU: Intensive Care Unit, SAPS: Simplified Acute Physiology Score, PaCO_2_: partial pressure of arterial CO_2_, MEAF: Model for Early Allograft Function, RIFLE: Risk Injury Failure Loss End-stage of kidney disease | | | |
