# Supplementary material for: Postoperative respiratory failure in liver transplantation: Risk factors and effect on prognosis
Source: PLoS One. 2019 Feb 11;14(2):e0211678. doi: 10.1371/journal.pone.0211678 (PMC6370207; doi:10.1371/journal.pone.0211678)
Supplement: S3 Table — The number of events (70) allowed the identification of 7 predictive factors. Hosmer-Lemeshow test: Chi2 = 3.78, p = 0.88). Age, MELD at transplant and pre-extubation PaCO2 showed a broad IQR (Interquartile Range) excursion, explaining how the low OR (Odd Ratio) is indicative of a strong statistical effect. MELD: Model for End-stage Liver Disease, VVBP: Veno-Venous bypass, PaCO2: partial pressure of arterial CO2, MEAF: Model for Early Allograft Function. (DOCX) [file pone.0211678.s004.docx]

**S3 Table. Details of the multivariate analysis (logistic regression)**

|  | | | | | | | | |  |
| --- | --- | --- | --- | --- | --- | --- | --- | --- | --- |
| **Factors** | **ß coefficient** | **SE** | **Wald** | **DF** | **P value** | **OR** | **95% Confidence Interval** | | |
| Age (recipient) | 0.046 | 0.018 | 6.61 | 1 | **0.010** | **1.05** | 1.01 | 1.09 | |
| Female sex | 1.026 | 0.433 | 5.63 | 1 | **0.018** | **2.79** | 1.19 | 6.52 | |
| MELD at transplant | 0.085 | 0.024 | 12.84 | 1 | **<0.001** | **1.09** | 1.04 | 1.14 | |
| Restrictive vs non-restrictive | 0.913 | 0.411 | 4.87 | 1 | **0.027** | **2.49** | 1.11 | 5.61 | |
| VVBP vs no-VVBP | 1.113 | 0.420 | 6.97 | 1 | **0.008** | **3.03** | 1.33 | 6.90 | |
| PaCO_2_ pre-extubation | 0.100 | 0.034 | 8.75 | 1 | **0.003** | **1.11** | 1.03 | 1.18 | |
| MEAF | 0.312 | 0.090 | 12.09 | 1 | **<0.001** | **1.37** | 1.15 | 1.63 | |
| *Intercept* | *-10.692* | *2.091* | *26.14* |  |  |  |  |  | |
|  | | | | | | | | | |

The number of events (70) allowed the identification of 7 predictive factors. Hosmer-Lemeshow test: Chi^2^ = 3.78 , p=0.88). Age, MELD at transplant and pre-extubation PaCO_2_ showed a broad IQR (Interquartile Range) excursion, explaining how the low OR (Odd Ratio) is indicative of a strong statistical effect. MELD: Model for End-stage Liver Disease, VVBP: Veno-Venous bypass, PaCO_2_: partial pressure of arterial CO_2_, MEAF: Model for Early Allograft Function.
