# Supplementary material for: Postoperative respiratory failure in liver transplantation: Risk factors and effect on prognosis
Source: PLoS One. 2019 Feb 11;14(2):e0211678. doi: 10.1371/journal.pone.0211678 (PMC6370207; doi:10.1371/journal.pone.0211678)
Supplement: S4 Table — PRF: Postoperative Respiratory Failure, BMI: body mass index, HCC: Hepatocellular carcinoma, MELD: Model for End-stage Liver Disease, LTx: Liver Transplantation, VVBP: Veno-Venous bypass, D-MELD: Donor Model for End-stage Liver Disease, BAR: BAlance of Risk score, CIT: Cold Ischemia Time, ICU: Intensive Care Unit, SAPS: Simplified Acute Physiology Score, PaO2: partial pressure of arterial oxygen, PaCO2: partial pressure of arterial CO2, FiO2: Fraction of Inspired Oxygen, MEAF: Model for Early Allograft Function, RIFLE: Risk Injury Failure Loss End-stage of kidney disease, LoS: Length of stay. (DOCX) [file pone.0211678.s005.docx]

| **Factors** | **Extubation failure (n=28)**  **Median (IQR) /Mean ± SD/n (%)** | **Weaning failure (n=44)**  **Median (IQR)/Mean±SD /n (%)** | ***P value*** |
| --- | --- | --- | --- |
| **PREOPERATIVE FACTORS (recipient)** |  |  |  |
| Age (years) | 58 (50-62) | 54 (47-62) | *0.90* |
| Female sex | 8 (28.6) | 14 (31.8) | *0.77* |
| BMI >30 | 2 (7.1) | 9 (20.5) | *0.13* |
| HCC | 11 (39.3) | 17 (38.6) | *0.96* |
| MELD at LTx | 20 (14-26) | 23 (15-31) | *0.06* |
| **MELDNa at LTx** | **23 (16-30)** | **27 (15-33)** | ***0.01*** |
| Encephalopathy grade ≥2 | 2 (7.1) | 2 (4.5) | *0.22* |
| Restrictive pattern | 7 (25.0) | 14 (31.8) | *0.56* |
| Obstructive pattern | 3 (10.7) | 3 (6.8) | *0.62* |
| **INTRAOPERATIVE factors** |  |  |  |
| Portal Vein thrombosis | 2 (7.1) | 7 (15.9) | *0.26* |
| **VVBP** | **5 (17.9)** | **18 (40.9)** | ***0.04*** |
| Packed red blood cell (units) | 11.3 ± 9.9 | 14.1 ± 11.3 | *0.24* |
| Packed red blood cell >10 units | 12 (42.9) | 28 (63.6) | *0.08* |
| Fresh Frozen Plasma (units) | 14.5 ± 14.8 | 22.0 ± 19.1 | *0.07* |
| Platelets (units) | 1.60 ± 1.80 | 1.75 ± 1.43 | *0.73* |
| Operation time (hours) | 12 (12-14) | 13 (12-14) | *0.58* |
| **LOGISTIC FACTORS** |  |  |  |
| D-MELD at LTx | 1067 ± 556 | 1172 ± 606 | *0.56* |
| BAR | 7.1 ± 4.0 | 8.8 ± 5.3 | *0.12* |
| CIT (hours) | 8 (7-8) | 8 (7-9) | *0.23* |
| **POST-OPERATIVE ICU FACTORS** |  |  |  |
| SAPS II at the ICU admission | 37.8 ± 13.3 | 41.6 ± 16.9 | *0.33* |
| PaO_2_ pre-extubation (mmHg) | 153.0 ± 31.8 | 139.0 ± 30.9 | *0.09* |
| PaCO_2_ pre-extubation (mmHg) | 37.4 ± 5.6 | 38.0 ± 6.1 | *0.68* |
| PaO_2_/FiO_2_ pre-extubation | 372 ± 88 | 337 ± 85 | *0.13* |
| **POST-OPERATIVE SURGICAL FACTORS** |  |  |  |
| MEAF | 5.3 ± 2.0 | 5.9 ± 1.9 | *0.23* |
| MEAF 8 & over | 3 (10.7) | 9 (20.5) | *0.28* |
| MELD on 3^rd^ p.o.d. (mg/dl) | 17.6 ± 6.2 | 18.9 ± 8.4 | *0.17* |
| Bilirubin on 3^rd^ p.o.d. (mg/dl) | 5.7 ± 4.3 | 8.0 ± 5.6 | *0.06* |
| Creatinine on 3^rd^ p.o.d. (mg/dl) | 1.32 ± 0.57 | 1.58 ± 0.73 | *0.10* |
| ***OTHER DATA (available after 48 hours)*** |  |  |  |
| ***PaO_2_ post-extubation (mmHg)*** | **89.0 ± 32.0** | **113.9 ± 31.6** | ***<0.01*** |
| *PaCO_2_ post-extubation (mmHg)* | 39.2 ± 7.8 | 39.3 ± 6.1 | *0.96* |
| ***PaO_2_/FiO_2_ post-extubation*** | **209 ± 93** | **290 ± 92** | ***<0.01*** |
| ***Mechanical Ventilation (hours)*** | **21 (14-39)** | **73 (66-119)** | ***<0.01*** |
| *Hemofiltration after extubation* | 1 (3.6) | 7 (15.9) | *0.10* |
| *Non-infectious lung involvement* | 17 (60.7) | 36 (81.8) | *0.22* |
| *Pneumonia* | 7 (25.0) | 13 (29.5) | *0.68* |
| ***Clavien-Dindo stratification*** |  |  |  |
| *Grade 0* | 3 (10.7) | 4 (9.1) | *0.82* |
| *Grade 1* | 9 (32.1) | 6 (13.6) | *0.06* |
| *Grade 2* | 7 (25.0) | 8 (18.2) | *0.49* |
| *Grade 3A* | 4 (14.3) | 3 (6.8) | *0.30* |
| *Grade 3B* | 3 (10.7) | 4 (9.1) | *0.82* |
| *Grade 4* | 1 (3.6) | 8 (18.2) | *0.07* |
| ***Grade 5*** | **1 (3.6)** | **11 (25.0)** | ***0.02*** |
| ***Grade 3B and higher*** | **5 (17.9)** | **23 (52.3)** | ***<0.01*** |
| *LoS in ICU post LTx (days)* | 9 (7-15) | 10 (7-18) | *0.81* |
| ***Death in ICU*** | **1 (3.6)** | **10 (22.7)** | ***0.03*** |
|  | | | |

**S4 Table**. **Characteristics of PRF patients according to extubation and weaning failure (univariate analysis)**

PRF: Postoperative Respiratory Failure, BMI: body mass index, HCC: Hepatocellular carcinoma, MELD: Model for End-stage Liver Disease, LTx: Liver Transplantation, VVBP: Veno-Venous bypass, D-MELD: Donor Model for End-stage Liver Disease, BAR: BAlance of Risk score, CIT: Cold Ischemia Time, ICU: Intensive Care Unit, SAPS: Simplified Acute Physiology Score, PaO_2_: partial pressure of arterial oxygen, PaCO_2_: partial pressure of arterial CO_2_, FiO_2_: Fraction of Inspired Oxygen, MEAF: Model for Early Allograft Function, RIFLE: Risk Injury Failure Loss End-stage of kidney disease, LoS: Length of stay.
